# Lessons learned from implementation of a national hotline for Ebola virus disease emergency preparedness in South Sudan

**DOI:** 10.1186/s13031-021-00360-x

**Published:** 2021-04-15

**Authors:** Velma K. Lopez, Sharmila Shetty, Angelo Thon Kouch, Matthew Tut Khol, Richard Lako, Alexandre Bili, Anyang David Ayuen, Agnes Jukudu, Ajak Ater Kug, Atem David Mayen, Emmanuel Nyawel, Kibebu Berta, Olushayo Olu, Kevin Clarke, Sudhir Bunga

**Affiliations:** 1 Division of Global Health Protection, Center for Global Health, CDC, Atlanta, Georgia USA; 2 Ministry of Health, Juba, South Sudan; 3 World Health Organization, Juba, South Sudan; 4 Division of Global HIV and TB, Center for Global Health, CDC, Juba, South Sudan

**Keywords:** Emergency preparedness, Ebola, Hotline

## Abstract

**Background:**

The world’s second largest Ebola outbreak occurred in the Democratic Republic of Congo from 2018 to 2020. At the time, risk of cross-border spread into South Sudan was very high. Thus, the South Sudan Ministry of Health scaled up Ebola preparedness activities in August 2018, including implementation of a 24-h, toll-free Ebola virus disease (EVD) hotline. The primary purpose was the hotline was to receive EVD alerts and the secondary goal was to provide evidence-based EVD messages to the public.

**Methods:**

To assess whether the hotline augmented Ebola preparedness activities in a protracted humanitarian emergency context, we reviewed 22 weeks of call logs from January to June 2019. Counts and percentages were calculated for all available data.

**Results:**

The hotline received 2114 calls during the analysis period, and an additional 1835 missed calls were documented. Callers used the hotline throughout 24-h of the day and were most often men and individuals living in Jubek state, where the national capital is located. The leading reasons for calling were to learn more about EVD (68%) or to report clinical signs or symptoms (16%). Common EVD-related questions included EVD signs and symptoms, transmission, and prevention. Only one call was documented as an EVD alert, and there was no documentation of reported symptoms or whether the person met the EVD case definition.

**Conclusions:**

Basic surveillance information was not collected from callers. To trigger effective outbreak investigation from hotline calls, the hotline should capture who is reporting and from where, symptoms and travel history, and whether this information should be further investigated. Electronic data capture will enhance data quality and availability of information for review. Additionally, the magnitude of missed calls presents a major challenge. When calls are answered, there is potential to provide health communication, so risk communication needs should be considered. However, prior to hotline implementation, governments should critically assess whether their hotline would yield actionable data and if other data sources for surveillance or community concerns are available.

## Background

Ebola virus outbreaks pose a recurring threat to global public health and have resulted in two WHO Public Health Emergency of International Concern declarations. The second largest Ebola outbreak in history occurred in the Democratic Republic of the Congo (DRC), with 3841 cases of Ebola virus disease (EVD) and 2299 deaths reported between 2018 and 2020 and spread to Uganda [1]. The largest known EVD outbreak occurred in West Africa between 2014 and 2016, resulting in 28,652 cases and 11,325 deaths [2]. Guinea [3], Liberia [4], and Sierra Leone [5] each implemented national telephone hotlines dedicated to EVD communication during the emergency response. Callers used the hotlines to report ill or dead individuals that may have had EVD and governments used this information to deploy rapid response teams to people in need. Lessons learned from the 2014-2016 outbreak emphasize the need for enhanced early warning [6, 7]. Rapid disease detection is, thus, a key component of emergency preparedness activities, particularly when a population is at high risk of an outbreak, and requires careful coordination with field-based health promotion and risk communication teams.

In the most recent DRC EVD outbreak, the risk for cross-border spread to South Sudan was very high. South Sudan experiences a high volume of population movements and cross-border travel from both DRC and Uganda [8–10]. Nascent health and public health infrastructure, ongoing conflict, insecurity, population displacement and poor infrastructure limit the country’s ability to rapidly respond and contain or limit an outbreak [11–13]. As South Sudan’s health infrastructure works to recover from a protracted complex humanitarian emergency, the impact can be severe if an Ebola outbreak is not detected and contained early. Thus, the World Health Organization (WHO) classifies South Sudan as a Priority 1 country for Ebola preparedness [14, 15]. In response, the South Sudan Ministry of Health (MoH) has scaled up Ebola preparedness activities, including the activation of a public health emergency operations center (PHEOC). Previous public health emergencies in South Sudan have been coordinated by interagency taskforces, and activation of the PHEOC marks an important step for emergency response capacity in South Sudan. One role of the PHEOC is to support EVD surveillance and response coordination. Given substantial geographic and infrastructure barriers to timely EVD case notification through existing surveillance and the limited access to regions at greatest risk for imported EVD cases, the MoH implemented a toll-free EVD hotline. The primary purpose of the hotline was to receive EVD alerts and augment surveillance, while its secondary goal was to provide evidence based EVD messages to the public. The dual use of a hotline in emergency preparedness for both surveillance and risk communication is a unique aspect of South Sudan’s EVD response.

The hotline allows communities to directly call the PHEOC to report unusual disease occurrences and/or symptomatology, potentially serving as a tool for rapid disease detection. Here, we describe use patterns of a national toll-free hotline in a complex public health operating environment to assess whether performance patterns were aligned with intended objectives. Specifically, we analyzed the hotline call logs in order to 1) evaluate call patterns to improve hotline operational performance, 2) assess why callers were using the hotline, and 3) provide recommendations to improve hotline performance, especially with regard to EVD surveillance and health communication. Lessons learned from this analysis have the potential to improve activities not only in ongoing emergency preparedness, but also for active emergency responses, such as for COVID-19.

## Methods

### EVD alert generation

The EVD hotline was implemented as the primary source to receive potential EVD notifications, or alerts, from communities, health facilities, designated EVD screening centres at points of entry, state and county surveillance officers, and any non-governmental partners working on EVD preparedness. If an alert was received, the PHEOC manager and the incident manager were notified and procedures were set in place to verify the alert immediately.

### Hotline operations

The hotline was staffed by PHEOC watch officers, i.e., MoH employees whose primary role was to record and communicate potential EVD cases to the response and surveillance team. The watch officers had cellular phones in their possession 24-h a day, 7 days a week, that function as the EVD hotline. The hotline operated centrally paid accounts with two of the major cellular network providers in the country, MTN and Zain, both of which are toll-free for callers. The MoH advertised the EVD hotline throughout the country through a variety of mechanisms and in multiple languages. For example, promotion of the hotline number (6666), was conducted through various risk communication platforms, including radio ads, poster campaigns, and community health workers. Messaging focused on calling the hotline if someone exhibited EVD-like symptoms or if a person died suddenly or inexplicably. Individuals were also encouraged to call the hotline to learn more about EVD.

Given the multiple objectives of the hotline, the watch officers received several rounds of training. First, they were trained on EVD surveillance operational procedures, including EVD alert and case definitions, documentation processes, and reporting processes. In addition, the watch officers were also trained by risk communication experts from UNICEF on common EVD myths and misconceptions.

### Hotline documentation and alert reporting

All received hotline calls were documented in free text on a paper form. Specifically, the following information from each call was recorded:

- the time and date of the call;
- the caller’s phone number, gender, and geographical administrative units (state, county, and *payam*);
- the reason for calling;
- the message given to the caller;
- the network that the call was received on;
- the name of the watch officer who took the call.

Approximately each week, these paper forms were entered into an Excel spreadsheet, which we refer to throughout the remainder of this text as a ‘call log’. Additionally, the total number of missed calls from each week was recorded at the top of the call log. No other information related to the missed calls was recorded. If an alert was received, the watch officer provided the caller’s phone number to the PHEOC manager for follow-up.

Following data entry, every 2 weeks, the watch officers provided a verbal update to the risk communication arm of the EVD response.

### Definitions

Received calls reflected calls in which the watch officer had a complete conversation with the caller. Missed calls had several meanings: a call that was dropped due to poor network, a received call where clearly articulated questions were not asked, a call that was not answered, or a “flashed call” (defined as when a caller dials the hotline number, allows it to ring once or twice, hangs up before the receiver can answer, and expects a call back).

### Analytical approach

We reviewed 41 call logs, with 22 call logs from the MTN network and 19 from the Zain network, from January 1 to June 8, 2019 (reflecting 22 weeks). Call logs were reviewed for completeness and we conducted a descriptive analysis on available data. Call log spreadsheets were merged to create one standard dataset. Responses to open-ended questions were cleaned and coded into thematic categories. Counts and percentages were calculated based on the specific variable of interest. R software, version 3.5.1, was used for all data cleaning and analysis.

The Human Research Protection Office within the Center for Global Health at the Centers for Disease reviewed the use of these data and determined it to be non-research secondary analysis.

## Results

**Fig. 1:**
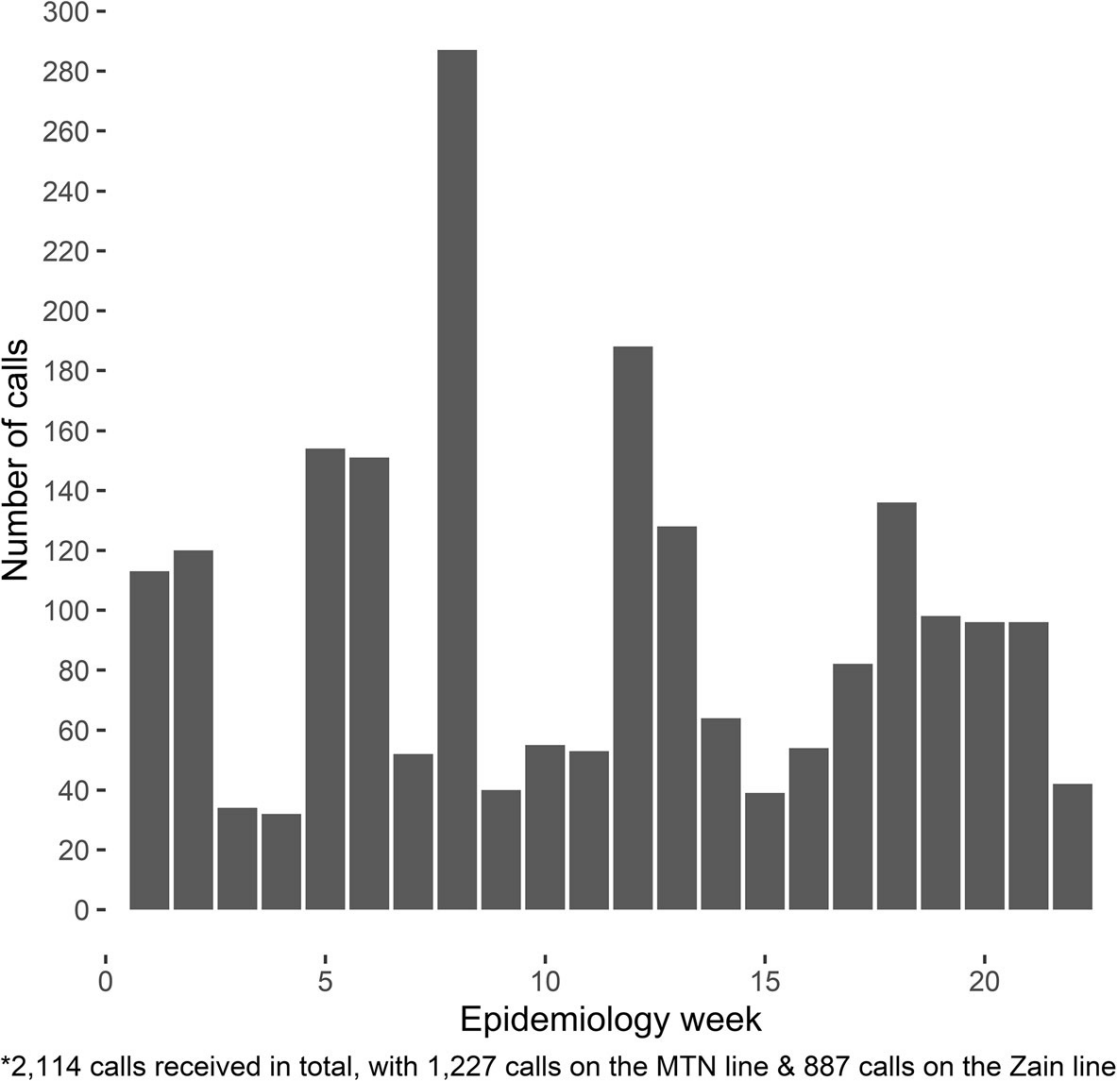
Number of received calls by epidemiology week (*n* = 2114*)

Missed calls (1835 calls in total) were documented on 17 of 41 call logs, while 2114 calls were received, with 58% of calls made to the MTN network and 42% through the Zain network (Fig. 1).

**Fig. 2:**
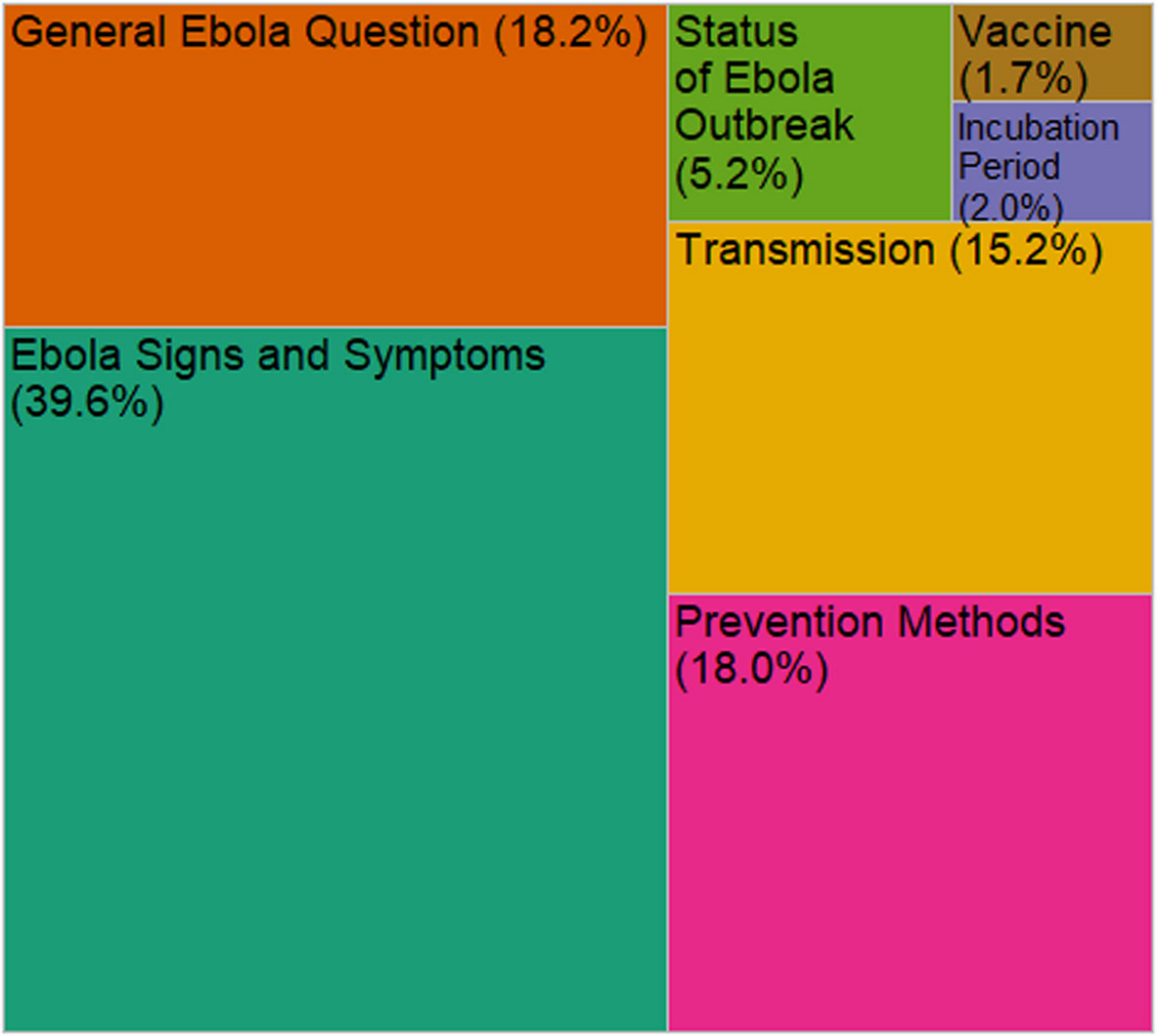
The percent distribution of Ebola-related queries into the hotline (*n* = 1431)

Callers used the hotline throughout the 24-h time period, with the median call time of received calls at 1:04 pm, and 7 days a week, with Thursday (16%), Fridays (16%), and Mondays (17%) being slightly more common than other days (11-14%). However, 45% of received calls were missing a date. Callers were most often men and individuals living in Jubek state, where the national capital Juba is located, while only 9.9% of calls were from border states that had the greatest risk of EVD introduction and also active EVD preparedness activities ongoing at the time of the analysis (Table 1). The leading reasons for calling the hotline were to learn more about EVD (68%) or to report a clinical sign or symptom (16%). Among those that used the hotline to learn more about EVD, 40 % wanted to know more about the signs and symptoms of EVD (Fig. 2). Other common reasons for calling included questions about EVD transmission, prevention, and general questions about EVD. An assessment of caller-reported clinical signs and symptoms alignment with the EVD case definition was not possible due to the free text format of the call log spreadsheet. Only one call was documented in the call log as an EVD alert, but there was no documentation of whether reported symptoms met the EVD case definition.

**Table 1:** Descriptive characteristics of hotline callers and reasons for calling the hotline. (*n* = 2114 received calls)

	Percent	Frequency
**Background Characteristics**		
**Female**	33.2	701
**State**		
Jubek^a^	34.1	721
Central Upper Nile	10.2	215
Wau^a^	8.8	186
Aweil	6.1	130
Torit	6.0	126
Gbudwe^a^	4.4	93
Bor	3.4	72
Renk	2.9	61
Bentiu	2.8	60
Yei River^a^	2.7	58
Yambio^a^	2.6	56
Rumbek	2.2	46
Tonj	1.4	29
Kuajok	1.3	28
Yirol	1.3	27
Eastern Lakes	1.2	26
Western Lakes	1.1	23
Gogrial	1.0	22
Kapoeta	1.0	22
Ruweng	1.0	22
Lol	0.9	18
Twic	0.7	15
Amadi	0.4	8
Melut	0.4	8
North Upper Nile	0.4	8
Warrab	0.3	6
Imatong	0.2	4
Maridi^a^	0.2	5
Mundri	0.2	4
Gok	0.1	2
Malakl	0.1	3
Terekeka	0.1	3
Upper Nile	0.1	3
Missing	0.2	4
**Reason for calling**		
To ask a question about Ebola	67.7	1431
To report a clinical sign or symptom	16.4	346
To ask a question about the hotline	3.8	81
To send a greeting to the MoH	1.8	37
To ask for money or airtime	1.7	36
To ask to join the illuminati	1.6	33
To ask for the radio to play a song	0.8	16
To ask about safety of eating bushmeat	0.4	8
Other Reason	5.7	120
Missing Reason	0.3	6

^a^ States with EVD preparedness activities

## Discussion

Traditionally, public health hotlines are implemented during an ongoing health emergency for public inquiry, passive surveillance, or response. For EVD specifically, hotlines have been historically used during active outbreak response. To our knowledge, the South Sudan hotline is the first documentation of a call-center in an outbreak preparedness phase. However, given only one EVD alert was generated from the hotline in this period, more effort is needed to reexamine data capture, as well as standard operating procedures for identifying alerts. Nevertheless, the hotline did show promise for augmenting risk communication activities. The high number of callers reporting clinical signs and symptoms and asking questions about EVD suggests that the MoH’s messaging about how to use the hotline was understood by callers. However, about 16% of calls were unrelated to EVD or other illnesses, highlighting an opportunity for additional messaging regarding hotline use. For example, watch officers reported prank calls and misconceptions about the hotline due to the phone number (6666), which are evident in the call logs. Additionally, the results highlight specific caller concerns related to EVD identification, transmission, and prevention. These themes can inform risk communication activities and empower communities to protect themselves and slow EVD spread in the event of an outbreak.

There are several potential novel uses for public health hotlines in infectious disease emergency preparedness. First, a hotline can provide real-time information to the public and allow open channels of communication with those responsible for preparedness activities; when accurate information is provided by an empathetic listener, there is an opportunity to increase community engagement and build trust. Examples of using social media within preparedness for these purposes have been previously encouraged [16], and within a low-resource setting, a hotline can serve the same purpose. Under conditions of good cellular networks and well-advertised hotlines, call volume may proxy for population engagement. Second, tailored risk communications or on-the-ground community engagement to geographical locations is possible based on call responses; this is particularly viable if misconceptions are recorded. Thus, a hotline can supplement rumor tracking. Real-time information, as opposed to a one-time survey, on community perceptions and misconceptions could help to better target behavior change communication. However, to implement rumor-tracking well, the watch staff will require continual training on how to effectively respond to these misperceptions and update them on new emerging issues. Such expanded activities require coordination with risk communication experts.

While our analysis provides insight into hotline use for surveillance and risk communcation, several limitations impact the analytical approach and interpretation. First, little information was available on missed calls. Although the documented number of missed calls rivals the number of received calls, missed calls were likely underestimated since less than half of all call logs recorded them. Additionally, because the missed calls were recorded in aggregate, it is not possible to further analyze distribution by hour of the day, day of the week, or watch officer responsible. Furthermore, the rationale for the missed call was not documented. Watch officers did not have funds to return missed calls, and the high volume makes returning calls impractical for the limited number of staff available. The PHEOC was not only missing data that could have been critical to the response, but may also have been losing important opportunities to communicate with the public and identify EVD alerts.

Second, the nature of free text data entry had several implications. Importantly, the reasons for calling were not categorized a priori, nor were categories determined by the watch officers. Consequently, imprecise information, coupled with post hoc categorization, may have resulted in misclassification of the reasons for calling into the hotline. Moreover, data were too limited to evaluate the quality of information the watch officers provided to callers, which is an important component of risk communication.

Finally, the data presented are not representative of the South Sudanese population. Although the hotline is a national number, cell phone coverage is inconsistent throughout the country, which influences which calls are received, dropped, and missed. For example, many of the states that border DRC where preparedness activities were ongoing have poor cellular network, and it is not clear whether these states are not using the hotline, or they simply cannot connect to the PHEOC.

## Conclusion

The identified limitations highlight opportunities to improve hotline data quality and utility of the hotline for early EVD detection and risk communication. Current standard operating procedures require that all EVD alerts be called into the hotline, however, only one call generated an EVD alert. Other EVD alerts have either come through the hotline without documentation or have been identified through informal channels. More effort is needed to not only document the how alerts were received by the MoH, but also to advertise hotline use to surveillance offices throughout the country, link the hotline-generated alerts with an EVD alert database, and to routinely review hotline information. Better documentation of missed calls is recommended, including documenting time and date, reason for missing the call, and, if a call was not returned, reasons why. This added specification will also improve tracking the workload of hotline staff. Development of a structured questionnaire to replace the open text format and using electronic data capture could reduce cumbersome data entry, missing information, and laborious data cleaning, and allow for more readily accessible data. Within the questionnaire, critical fields should include components of the EVD case definition and whether the call generated an EVD alert. Additionally, routine analyses of hotline calls may augment risk communications data collected from community health works and other field-based staff. This is particularly important in contexts where a hotline serves as a data source for community concerns. Nevertheless, integration of surveillance and risk communication requires strong coordination between different components of outbreak response teams. These findings will be useful for improving the hotline, both in its current function for COVID response in South Sudan, as well as to other countries that might consider using hotlines for similar preparedness efforts.

## Data Availability

The datasets generated and analyzed are not publicly available, as they are part of national health surveillance by the government of South Sudan.

## Abbreviations

DRC: Democratic Republic of Congo
MoH: Ministry of Health
EVD: Ebola virus disease
WHO: World Health Organizations
PHEOC: Public Health Emergency Operations Center
